# LZTS2 and PTEN collaboratively regulate ß-catenin in prostatic tumorigenesis

**DOI:** 10.1371/journal.pone.0174357

**Published:** 2017-03-21

**Authors:** Eun-Jeong Yu, Erika Hooker, Daniel T. Johnson, Mi Kyung Kwak, Kang Zou, Richard Luong, Yongfeng He, Zijie Sun

**Affiliations:** 1 Department of Cancer Biology, Beckman Research Institute, City of Hope, Duarte, California, United States of America; 2 Department of Urology, Stanford University School of Medicine, Stanford, California, United States of America; 3 Department of Comparative Medicine, Stanford University School of Medicine, Stanford, California, United States of America; University of Alabama at Birmingham, UNITED STATES

## Abstract

The *leucine zipper tumor suppressor 2 (LZTS2)* was identified as a tumor susceptibility gene within the 10q24.3 chromosomal region, and is approximately 15Mb from the *PTEN* locus. This region containing the both loci is frequently deleted in a variety of human malignancies, including prostate cancer. LZTS2 is a ß-catenin-binding protein and a negative regulator of Wnt signaling. Overexpression of PTEN in prostate cancer cell lines reduces ß-catenin-mediated transcriptional activity. In this study, we examined the collaborative effect of PTEN and LZTS2 using multiple *in vitro* and *in vivo* approaches. Co-expression of PTEN and LZTS2 in prostate cancer cells shows stronger repressive effect on ß-catenin mediated transcription. Using a newly generated mouse model, we further assessed the effect of simultaneous deletion of *Pten* and *Lzts2* in the murine prostate. We observed that mice with both *Lzts2* and *Pten* deletion have an earlier onset of prostate carcinomas as well as an accelerated tumor progression compared to mice with *Pten* or *Lzts2* deletion alone. Immunohistochemical analyses show that atypical and tumor cells from compound mice with both *Pten* and *Lzts2* deletion are mainly composed of prostate luminal epithelial cells and possess higher levels of cytoplasmic and nuclear β-catenin. These cells also exhibit a higher proliferative capacity than cells isolated from single deletion mice. These data demonstrate the significance of simultaneous *Pten* and *Lzts2* deletion in oncogenic transformation in prostate cells and implicates a new mechanism for the dysregulation of Wnt/β-catenin signaling in prostate tumorigenesis.

## Introduction

The leucine zipper tumor suppressor 2 (LZTS2), also called Lapser1, was originally identified based on homology with the LZTS1 tumor suppressor [1]. Lzts2 null mice showed no obvious pre- or post-natal lethality, but a portion of the mice developed defects in the kidney and urinary tract, including renal/ureteral duplication, hydroureter, and hydronephrosis [2]. Aged Lzts2 null mice also presented with increased spontaneous tumor development [3]. When treated with N-butyl-N-(4-hydroxybutyl) nitrosamine (BBN), both homozygous and heterozygous *Lzts2* deletion mice showed increased susceptibility to urinary bladder carcinoma development [3]. LZTS2 has also been shown to interact with ß-catenin [4]. A Rev-like leucine-rich, CRM1/exportin-regulated nuclear export signal (NES) sequence was identified within the carboxyl terminal region of LZTS2. Through this NES site, LZTS2 can modulate the export of nuclear ß-catenin, reducing the transcriptional activity of ß-catenin in the cell [4]. These data suggest that LZTS2 is a bona fide regulator of ß-catenin and plays critical role in development and tumorigenesis.

The tumor suppressor PTEN is a phosphoprotein/phospholipid dual-specificity phosphatase [5]. Somatic mutation of *PTEN* frequently occurs in a variety of human tumors, including prostate cancer [6]. It has been shown that PTEN inhibits the activity of AKT/PKB, a key effector of the phosphatidylinositol 3-kinase (PI3K) signaling pathway, and functions as a tumor suppressor [7]. Activation of AKT can phosphorylate a number of downstream substrates, including glycogen synthase kinase 3ß, GSK3ß, [8,9]. Loss of PTEN increases GSK3ß phosphorylation and results in inhibiting ß-catenin degradation through the destruction complex [10].

Deletion of the human chromosomal 10q23-24 has been frequently observed in many human tumors, including prostate cancer. *PTEN* was identified within 10q23.3 region [11,12], and *LZTS2* is located at 10q24.3, approximately 15Mb from the *PTEN* locus [1]. Intriguingly, both 10q23.3 and 10q24.3 regions, containing *PTEN* and *LZTS2*, are frequently deleted in a variety of human tumors [1,13]. *PTEN* deletion is closely associated with prostate cancer initiation and progression [6]. LZTS2 is expressed in human testis, prostate, and ovary tissues [4], and reduced expression of LZTS2 transcripts and proteins has been observed in prostate cancer tissues [3]. Similar to humans, in the mouse, *Lzts2* is located on chromosome 19, only 11Mb from the mouse *Pten* gene [14]. In this study, we observed that PTEN and LZTS2 collaboratively enhance the transcriptional activity of ß-catenin in prostate cancer cells. To fully investigate the collaborative role of PTEN and LZTS2 in prostate tumor development, we generated a mouse model, in which both floxed *Pten* and *Lzts2* alleles were targeted on chromosome 19. We subsequently crossed this mouse line with *Probasin-Cre4* mice [15], and generated *Lzts2*^*LoxP/LoxP*^:*PB-Cre4*, *Pten*^*loxP/Wt*^:*PB-Cre4*, and *Lzts2*^*LoxP/LoxP*^ *-Pten*^*loxP/Wt*^:*PB-Cre4* mice. Using these mouse models, we characterized the biological consequences of the loss of either or both Pten and Lzts2 in the mouse prostatic luminal epithelium. We detected increased cellular proliferation in the prostates of *Lzts2*^*LoxP/LoxP*^ *Pten*^*loxP/Wt*^:*PB-Cre4* compound mice, and observed accelerated tumor development and aggressive tumor invasion. These data elucidate a collaborative role of loss of both Pten and Lzts2 in prostate tumorigenesis, and implicate a critical role of Wnt/ß-catenin in prostate tumorigenesis.

## Experimental procedures

### Cell cultures and transfections

Human prostate cancer cell lines, PC3 and DU145, were maintained in DMEM supplemented with 5% fetal calf serum (FCS) (HyClone, Denver, CO). An AR-positive prostate cancer cell line, LNCaP, was maintained in T-medium (Invitrogen, Carlsbad, CA) with 5% FCS. Transient transfections were carried out using a LipofectAMINE transfection kit or LipofectAMINE 2000 (Invitrogen, Carlsbad, CA).

### DNA plasmids, and luciferase and ß-galactosidase assays

TOPflash (pGL3-OT) and FOPflash luciferease (pGL3-OF) reporters were obtained from Dr. Bert Vogelstein (Johns Hopkins University School of Medicine, Baltimore, MD). A CMV-driven ß-galactosidase (ß-gal) reporter was generated by cloning the lacZ gene into the pcDNA3 vector [16]. The pcDNA-Tcf4 construct was provided by Dr. H. C. Clevers (CBG, Utrecht, The Netherlands). Expression constructs of human PTEN were generously provided by Dr. William Sellers (Dana-Farber Cancer Institute, Boston, MA). The full-length cDNA of human ß-catenin was cloned into pCDNA3 expression vector and mutants of ß-catenin with a single point mutation in the GSK3ß phosphorylation sites were generated by a PCR-based mutagenesis scheme as described previously [16]. LZTS2 expression vectors and shRNA pLentiviral vectors were generated as previously described [2,4].

Luciferase activity was measured in relative light units (RLU) as previously described [2,4,16,17]. Briefly, 50 μl of cell lysate was used for luciferase assays. The light output is measured after a 5 sec delay following injection of 50 μl luciferase buffer and 50 μl luciferin by the dual injector luminometer, according to the manufacturer’s instructions (Analytical Luminesence Lab., San Diego, CA). The RLU from individual transfections were normalized by measurement of ß-galactosidase activity expressed from a co-transfected plasmid. Individual transfection experiments were done at least three times in triplicate and the results are reported as mean luciferase/ß -galactosidase (±SD) from representative experiments.

### Mouse mating and genotyping

We have previously generated a floxed allele for the mouse *Lzts2* gene on chromosome 19 [2]. Mice homozygous for floxed *Pten* exon 5, *Pten*^*loxP/loxP*^, were obtained from the Jackson Laboratory (Strain#: 004597, Bar Harbor, ME). We then intercrossed *Lzts2*^*LoxP/+*^ with *Pten*^*loxP/LoxP*^ mice to generate *Lzts2*^*LoxP/+*^-*Pten*^*loxP/LoxP*^ compound mice through homologous recombination. To make the prostate specific conditional knockout mouse line, we bred the above mice with *PB-Cre4* mice [15] to generate *Lzts2*^*LoxP/LoxP*^:*PB-Cre4*, *Pten*^*loxP/Wt*^:*PB-Cre4*, and *Lzts2*^*LoxP/LoxP*^*-Pten*^*loxP/Wt*^:*PB-Cre4* mice in this study.

Genomic DNA samples isolated from mouse tail tips or embryo yolk sacs were used for genotyping as described in our previous reports [2,18]. Three primers were used to identify wild type and *Lzts2* deleted alleles, including common forward primer, 5′-TACCATCTGAGTTGCTGATTGC-3′; wild type reverse primer, 5′-AGAGAGGAAGGAATGGGAGATC-3′; deleted reverse primer, 5′-CACAAGGAATGCTCCAACCCTG-3′. PCR was performed as follows: 5 min 94°C and then 35 cycles of 94°C for 45 sec, 60°C for 45 sec, and 72°C for 80 sec, followed by a final step at 72°C for 10 min. For *Pten* allele, we used the forward primer (5’-TCCCAGAGTTCATACCAGGA-3’) and the reverse primer (5’-AATCTGTGCATGAAGGGAAC -3’) to distinguish the wild type and target alleles by amplifying the flanking *loxP* sites. For detection of deleted exon 5, the forward primer 5’-ACTCAAGGCAGGGATGAGC-3’, and reverse primer, 5-GCTTGATATCGAATTCCTGCAGC-3’ were used [19]. The forward primer 5’GATCCTGGCAATTTCGGCTAT-3’ and reverse primer 5’GCAGGAAGCTACTCTGCACCTTG-3’ were used to detect the *PB*-*Cre* transgene. Genomic DNA fragments were amplified at 95°C for 5 min, then 95°C for 45 sec, 58°C for 40 sec, and 72°C for 60 sec for 36 cycles, then 72°C for 5 min. We made littermate controls lacking the *Cre* transgene in all experiments. All animal experiments performed in this study were approved by the ethics committee of the Administrative Panel on Institutional Animal Care and Use Committee at Stanford University and Beckman Research Institute/City of Hope, respectively.

### Histological analyses and immunohistochemistry

In this study, we used the new guidelines recommended by The Mouse Models of Human Cancers Consortium Prostate Pathology Committee in 2013 for our pathological analyses [20]. Mouse tissues were fixed and processed as described in our previous study [21]. Slides were subsequently counterstained with 5% (w/v) Harris hematoxylin. For histological analysis, 5-μm serial sections were processed from xylene to water through a decreasing ethanol gradient, stained with hematoxylin and eosin, and processed back to xylene through an increasing ethanol gradient. For immunohistochemical assays, 5-μm sections were boiled in 0.01M citrate buffer (pH 6.0) or Tris-EDTA-Tween (pH 9.0) for 20 mins after re-dehydration from xylene to water, and blocked by 5% goat serum. Tissue sections were then incubated with 1:500 dilution of anti-mouse/human AR (Rabbit polyclonal Ab, Santa Cruz, sc-816), 1:100 anti-Pten (Rabbit mAb, Cell Signaling, 9559), 1:300 dilution of anti-p63(Rabbit polyclonal Ab, Santa Cruz, sc-8343), 1:3000 of anti-Ki67(Mouse mAb, Novacastsra, NCL-ki67), 1:300 of anti-E-cadherin(Mouse mAb, BD Transduction Laboratories, c20820), 1:1000 of anti-K5 (Rabbit polyclonal, Covance, PRB-160P), 1:1000 of anti-K8 (Mouse mAb, Covance, MMS-162P), 1:200 of synaptophysin antibody (Rabbit polyclonal, Invitrogen, 180130), 1:500 of anti-ß-catenin (Mouse mAb, BD Transduction Laboratories, 610154) or an “in-house” rabbit polyclonal anti-Lzts2 antibody [2], in 1% of goat serum at 4°C overnight. Tissues were incubated with biotinylated goat anti-mouse or goat anti-rabbit (Vector Laboratories, BA-1000 or BA-9200) at 1:1000 dilution for 1 hr at room temperature followed by a 30 min incubation with horse radish peroxidase (HRP)-conjugated streptavidin (Vector Laboratories, SA-5004). Immunostainings were visualized using DAB kit (Vector Laboratories, SK-4100). Images for all HE and immunohistochemistry experiments in this study were acquired on a Leica dissecting microscope (model MZ9_5_) using Zeiss Axiovision software.

### Preparation of whole cell lysates and nuclear extracts, and immunoprecipitation and blotting

Different aged mouse embryos were cut into small pieces, homogenized, and then used for making both cytosolic and nuclear extracts as described previously [10,16]. The cytosolic fractions were prepared in digitonin lysis buffer (1% digitonin, 150 mM NaCl, 50 mM Tris-HCl pH 7.5, 10 mM MgCl2) or in RIPA buffer (0.5% Nonidet P-40, 0.3% Triton X-100, 15mM MgCl2, 5mM EDTA, 150mM NaCl, 50mM Tris-HCl pH 7.8), respectively [22]. Nuclear extracts were prepared as described previously [23].

Protein fractions for immunoblotting were boiled in SDS-sample buffer and then resolved on a 10% SDS-PAGE. The proteins were transferred onto a nitrocellulose membrane and probed with anti-ß-catenin antibody (Santa Cruz Biotechnology), anti-tubulin (clone DM1A, Neomarker), PCNA (PC10, Termo Fisher Scientific), or the polyclonal Lzts2 antibody [2]. Proteins were detected using the ECL kit (Amersham, Arlington Heights, IL). The antibody against tubulin (Neomarker, Fremont, CA) was used for protein loading.

### Statistical analyses

We presented the data as the mean ±SD. We made comparisons between groups, using a two-sided Student’s *t* test. P<0.05 and P<0.01 were considered significant.

## Results

### PTEN expression regulates ß-catenin transcriptional activity

**Fig 1:**
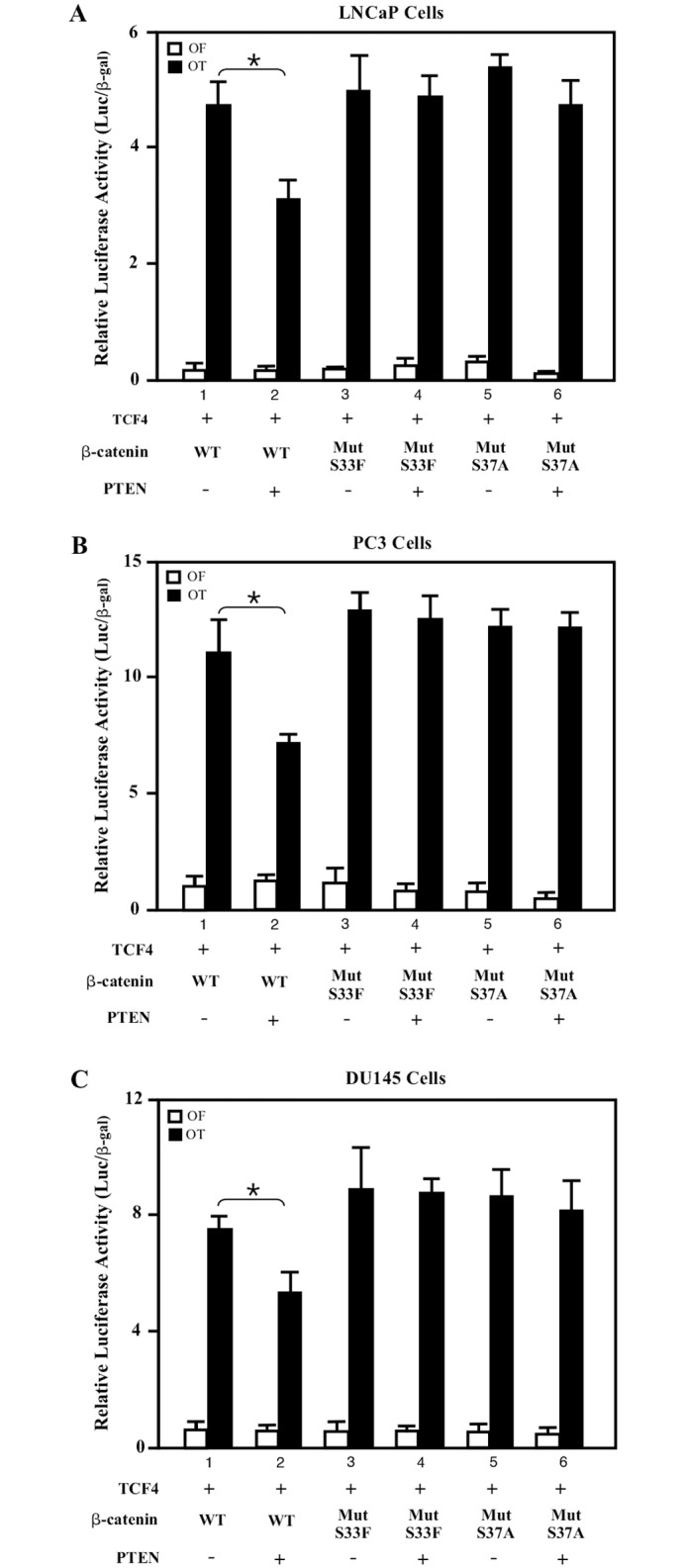
PTEN represses ß-catenin transcriptional activity in multiple prostate cancer cell lines. (A) LNCaP cells were transfected with TOPflash (pGL3-OT) or FOPflash (pGL3-OF) luciferase reporter (100 ng), pcDNA3-ß-gal (25 ng), pcDNA3-Tcf4 (5 ng), and the wild-type or mutants of pcDNA3-Flag-ß-catenin (50 ng). Either an empty pCMV5 vector or pCMV5-PTEN were co-transfected with the above plasmids. Cell lysates were measured for luciferase and ß-gal activities. Similar experiments were performed in (B) PC-3 and (C) DU-145 cells. The data represent the mean ± S.D. of three independent samples. “*” means P<0.05.

It has been shown that wild-type PTEN expression inhibits the enhancement of ß-catenin mediated transcriptional activity in prostate cancer cells [10]. LZTS2 has also been shown to interact with ß-catenin and modulate the export of nuclear ß-catenin, reducing the transcriptional activity of ß-catenin [4]. In addition, both human and murine *Pten* and *Lzts2* genes are closely localized on chromosome 10 or 19, respectively [14,24]. Furthermore, the deletion of both 10q23.3 and 10q24.3 regions that contain *PTEN* and *LZTS2* genes have been frequently observed in a variety of human tumors [1,13]. Therefore, based on these lines of evidence, we examined the collaborative effect of PTEN and LZTS2 in regulating ß-catenin activity. We performed transient transfections in several prostate cancer cell-lines using either wild-type or stabilized mutant ß-catenin to assess PTEN expression in ß-catenin mediated transcription. These ß-catenin mutants contain point mutations within the phosphorylation site of GSK3β (S33F or S37A), which prevents degradation via the ubiquitin proteasome pathway. As shown in Fig 1, co-expression of TCF4 and ß-catenin induced transcription of the TOPflash (pGL3-OT) reporter in all three prostate cancer lines, including LNCaP, PC3, and DU145 (Fig 1A, 1B and 1C). Interestingly, a significant reduction of ß-catenin mediated transcriptional activity was observed when a wild-type PTEN vector was co-transfected with the wild-type ß-catenin expression vectors in all of three different cell lines (see lines 1 versus lines 2 in Fig 1A–1C, P<0.05). In contrast, there is almost no change in samples co-transfected with either stabilized mutant ß-catenin vectors in the presence or absence of PTEN (lines 3 to 6, Fig 1A–1C). The well described ß-catenin mutants used above are impervious to degradation by the destruction complex [25,26]. Therefore, these results suggest that PTEN can negatively regulate ß-catenin-mediated transcription in a GSK3ß-dependent manner.

### PTEN and LZTS2 collaboratively regulate β-catenin transcriptional activity

**Fig 2:**
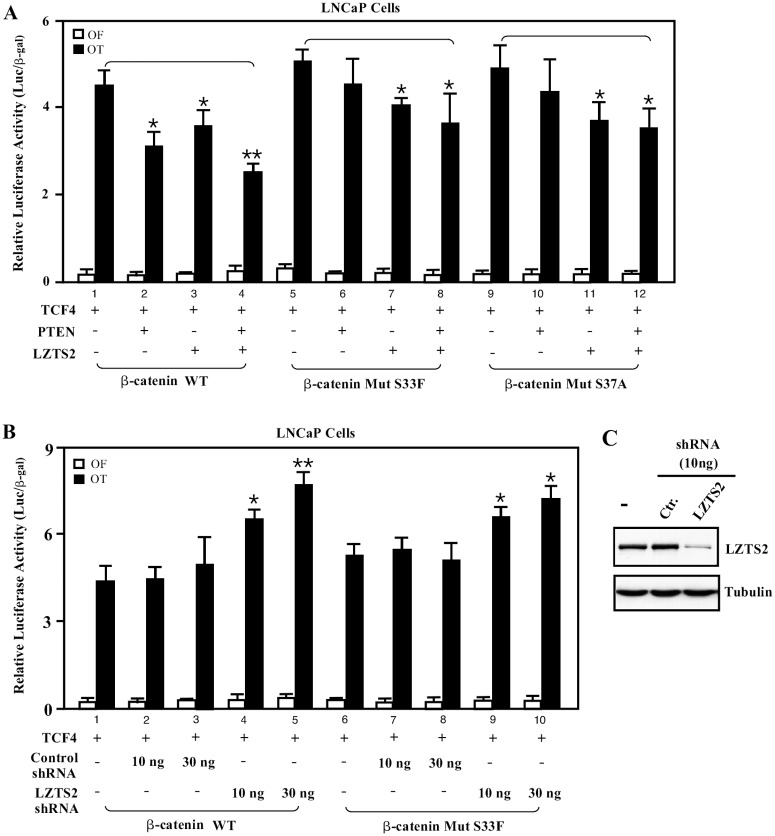
LZTS2 and PTEN collaboratively repress β-catenin-mediated transcription. (A) One hundred ng of pGL3-OT (OT) or pGL3-OF (OF), 25 ng of pcDNA3-ß-gal, 5 ng of TCF4 expression vector, and 50 ng of wild-type or mutant ß-catenin were co-transfected with either pcDNA3-FLAG-hLZTS2 or pCMV5-PTEN plasmids alone or together into LNCaP cells. Cell lysates were measured for luciferase and ß-gal activities. (B) LNCaP cells were transfected with either 100 ng of pGL3-OT (OT) or pGL3-OF (OF), 25 ng of pcDNA3-ß-gal, 50 ng of wild-type or mutant ß-catenin, and control or Lzts2-targeted shRNA as indicated. Luciferase and ß-gal activities were measured as described above. The data represent the mean ± S.D. of three independent samples. (C) LNCaP cells were transfected with either control or LZTS2 targeted shRNAa representative western blot with antibodies against human LZTS2 or Tubulin is shown. “*” or “**” means P<0.05 or <0.01, respectively.

Next, we examined the possible collaborative effect of PTEN and LZTS2 on ß-catenin-mediated transcription. Co-expression of TCF4 and ß-catenin showed a transcriptional induction of pGL3-OT in LNCaP cells (Fig 2A). Transfection of PTEN or LZTS2 alone repressed wild type ß-catenin mediated transcriptional activity (lines 2 and 3, Fig 2A), while co-transfection of both PTEN and LZTS2 displayed significantly stronger repression (p<0.01, line 4 versus line 1, Fig 2A). In contrast, LZTS2 expression showed a repression on pGL3-OT promoter/reporter mediated by both wild type and mutated ß-catenin (lines 7, 8, 11, and 12, Fig 2A). These data suggest that LZTS2 can repress ß-catenin mediated transcription collaboratively with PTEN, and its regulatory mechanism of ß-catenin is distinct from PTEN-mediated repression [4]. We then evaluated the repressive effect of endogenous LZTS2 using short hair-pin RNA (shRNA) interference. Transfection of LZTS2 shRNA, but not control shRNA, showed reduced expression of endogenous LZTS2 proteins in LNCaP cells (Fig 2C). These knockdown effects also resulted in a dosage-dependent activation of both wild type and stabilized mutant ß-catenin with mutations of the serine residues on the pGL3-OT promoter/reporter in LNCaP cells (Fig 2B). In contrast, there is no change in samples transfected with the control shRNA vector, suggesting that the above effect was due to LZTS2 knock-down. Taken together, these data demonstrate the role of LZTS2 in the regulation of ß-catenin-mediated transcription.

### Generation of the *Lzts2* and *Pten* compound mice

**Fig 3:**
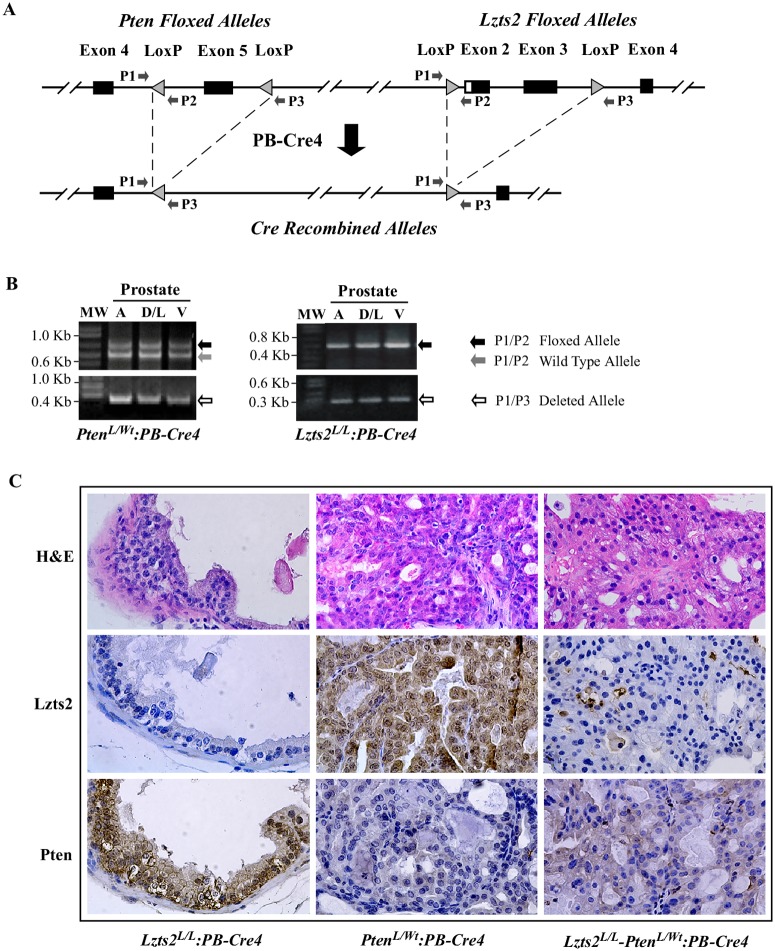
Generation of mice with prostate-specific *Lzts2* and *Pten* deletion. (A) Schematic representation of *Lzts2* and *Pten* compound mice. On chromosome 19, loxP sites flank exon 5 of the *pten* gene and exons 2 and 3 of the *lzts2* gene. Crossing with *PB-Cre* mice results in prostate-specific recombination of the loxP sites and removal of these exons from this locus. (B) Genomic PCR analysis of *Lzts2* and *Pten* alleles in prostate tissues using specific primers as detailed in (A). (C) Histological and immunohistochemical analysis of 6–8 month old *Lzts2*^*LoxP/LoxP*^:*PB-Cre4*, *Pten*^*loxP/Wt*^:*PB-Cre4*, and *Lzts2*^*LoxP/LoxP*^ *-Pten*^*loxP/Wt*^:*PB-Cre4* mice. Top panel depicts H&E staining of prostate tissues. Immunohistochemistry of Lzts2 (middle) and Pten (lower) on sequential sections are shown below.

To further examine the collaborative role of PTEN and LZTS2 *in vivo*, we took a loss-of-function approach to directly address the biological significance of PTEN and LZTS2 in tumorigenesis using an *Lzts2* and *Pten* deficient mouse strain. Because murine *Lzts2* is located approximately 11Mb away from *Pten* [14], we recombined floxed *Pten* and *Lzts2* loci into chromosome 19 by crossing *Pten* and *Lzts2* floxed mice [2,19]. To examine the role of Pten and Lzts2 in the murine prostate, we subsequently crossed this mouse model with Probasin-Cre4 mice [15], and generated *Lzts2*^*LoxP/LoxP*^:*PB-Cre4*, *Pten*^*loxP/Wt*^:*PB-Cre4*, and *Lzts2*^*LoxP/LoxP*^ *-Pten*^*loxP/Wt*^:*PB-Cre4* mice (Fig 3A). Using specific primers (Fig 3A), we assessed mouse genotypes using genomic PCR analysis. We observed both appropriate floxed and deleted *Pten* and *Lzts2* alleles in mouse prostate tissues (Fig 3B). We then evaluated Pten and Lzts2 expression in prostate tissues, which were isolated from 6–8 month old mice with different genotypes, using immunohistochemistry. As shown in Fig 3C, Lzts2 staining was observed in prostatic luminal cells of *Pten*^*loxP/Wt*^:*PB-Cre4* mice, but very low or no staining with Lzts2 antibody was detected in samples isolated from *Lzts2*^*LoxP/LoxP*^:*PB-Cre4* and *Lzts2*^*LoxP/LoxP*^ *-Pten*^*loxP/Wt*^:*PB-Cre4* mice. In a similar vein, decreased staining with a Pten specific antibody was observed in prostate tissue samples isolated from both *Pten*^*loxP/Wt*^:*PB-Cre4* and *Lzts2*^*LoxP/LoxP*^ *-Pten*^*loxP/Wt*^:*PB-Cre4* mice. These data demonstrate that either or both *Lzts2* and *Pten* are deleted in the prostate of *Lzts2*^*LoxP/LoxP*^:*PB-Cre4*, *Pten*^*loxP/Wt*^:*PB-Cre4*, and *Lzts2*^*LoxP/LoxP*^ *-Pten*^*loxP/Wt*^:*PB-Cre4* mice, respectively.

### Conditional deletion of Lzts2 accelerates Pten-mediated oncogenic transformation in the mouse prostate

**Fig 4:**
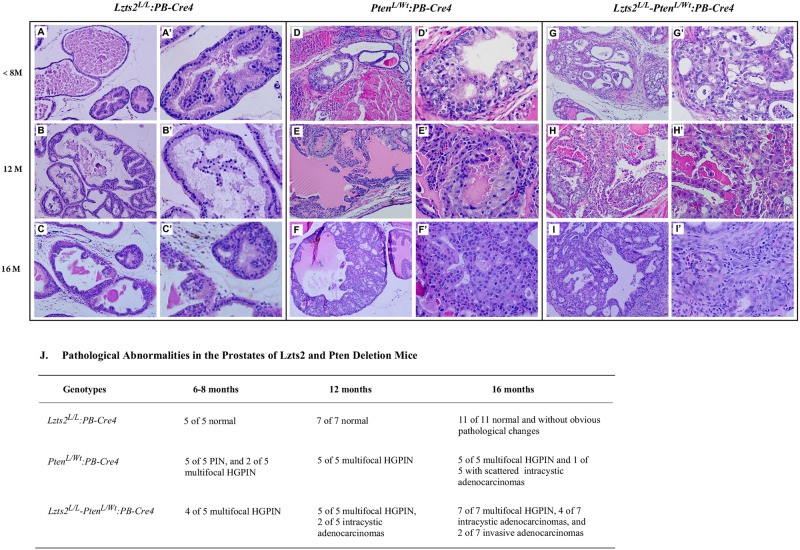
Prostate-specific *Lzts2* deletion accelerates Pten-mediated tumorigenesis. Prostates from mice between the ages of 2 to 16-months were analyzed for neoplastic lesions following H&E staining as per the guidelines from the Mouse Models of Human Cancers Consortium Prostate Pathology Committee. Panels depict 10x images (A-I) and 40x images (A’-I’) of *Lzts2*^*LoxP/LoxP*^:*PB-Cre4* (panels A-C’), *Pten*^*loxP/Wt*^:*PB-Cre4* (panels D-F’), and *Lzts2*^*LoxP/LoxP*^ *-Pten*^*loxP/Wt*^:*PB-Cre4* (panels G-I’) mice. (J) Table describing pathological abnormalities in cohorts of aged *Lzts2*^*LoxP/LoxP*^:*PB-Cre4*, *Pten*^*loxP/Wt*^:*PB-Cre4*, and *Lzts2*^*LoxP/LoxP*^ *-Pten*^*loxP/Wt*^:*PB-Cre4* mice.

*Lzts2*^*LoxP/LoxP*^ *-Pten*^*loxP/Wt*^:*PB-Cre4* compound mice as well as *Lzts2*^*LoxP/LoxP*^:*PB-Cre4* and *Pten*^*loxP/Wt*^:*PB-Cre4* mice were born at the expected Mendelian ratios and appeared normal with no obvious differences from their wild-type littermates at birth. We systematically examined male mice starting at 2-months of age and followed them until at least 16-months of age. We did not observe obvious abnormalities in 16 to 22-month-old *Lzts2*^*LoxP/LoxP*^: *PB-Cre4* mice (Fig 4A–4C’). Adhering to recommendations of the Mouse Models of Human Cancers Consortium Prostate Pathology Committee [20], we observed the development of prostatic intraepithelial neoplasia (PIN) in 6-month-old *Pten*^*loxP/Wt*^:*PB-Cre4* mice. The PIN lesions first occurred in ventral prostate (VP), and then extended to dorsal (DP), lateral (LP), and anterior (AP) lobes. With time, these mPIN lesions progressed towards high-grade mPIN lesions or prostatic intracystic adenocarcinomas (Fig 4E–4F’). These lesions originated predominantly in the dorsal/lateral prostate (D/LP) and ventral prostate (VP) lobes, which is consistent with previous observations [19]. Notably, more *Lzts2*^*LoxP/LoxP*^ *-Pten*^*loxP/Wt*^:*PB-Cre4* compound mice developed HGPIN lesions at 6-months of age than *Pten*^*loxP/Wt*^:*PB-Cre4* mice (Fig 4G and 4G’). The compound mice also showed accelerated tumor development. At 12-months of age, half of the *Lzts2*^*LoxP/LoxP*^ *-Pten*^*loxP/Wt*^:*PB-Cre4* mice developed prostatic intracystic adenocarcinomas, and at 16-months, almost all of the *Lzts2*^*LoxP/LoxP*^ *-Pten*^*loxP/Wt*^:*PB-Cre4* mice progressed to prostatic intracystic adenocarcinomas (Fig 4J). Using the Fisher's exact test, we analyzed the difference in prostatic adenocarcinoma formation between *Pten*^*loxP/Wt*^:*PB-Cre4* and *Lzts2*^*LoxP/LoxP*^ *-Pten*^*loxP/Wt*^:*PB-Cre4* mice in 12 and 16 age groups, and observed a significant difference (P<0.05). These results clearly demonstrate that deletion of *Lzts2* accelerates prostate tumor progression in *Pten*^*loxP/Wt*^:*PB-Cre4* mice.

### Identifying cellular origins of atypical and tumor cells

**Fig 5:**
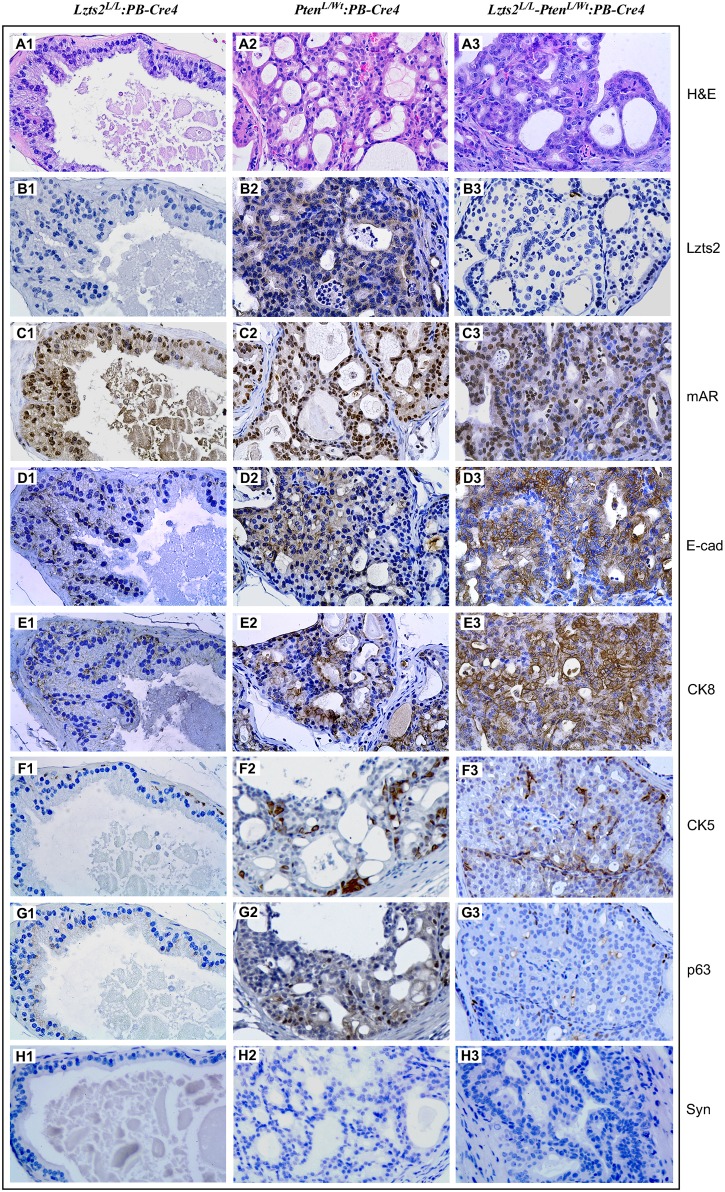
Mouse PINs from *Lzts2*-*Pten* compound mice are composed primarily of luminal epithelial cells. Immunohistochemical comparison of prostates from *Lzts2*^*LoxP/LoxP*^:*PB-Cre4* (panel 1), *Pten*^*loxP/Wt*^:*PB-Cre4* (panel 2), and *Lzts2*^*LoxP/LoxP*^ *-Pten*^*loxP/Wt*^:*PB-Cre4* (panel 3) mice. Prostates were stained with H&E (Panels A1-A3) for histological comparison and Lzts2 (B1-B3), mouse androgen receptor (C1-C3), E-cadherin (D1-D3), cytokeratin 8 (E1-E3), cytokeratin 5 (F1-F3), p63 (G1-G3), and Synaptosin (H1-H3) to characterize the atypical cells.

Mouse prostatic epithelium is composed of several cell types, including basal and luminal epithelial cells, as well as neuroendocrine cells. Previous studies have shown that luminal epithelial cell markers have been detected in PIN and prostatic adenocarcinoma lesions in *Pten* prostate conditional knockout mice with *ARR2PB-Cre* [19]. To determine the cellular origin of PIN in *Lzts2*^*LoxP/LoxP*^ *-Pten*^*loxP/Wt*^:*PB-Cre4* compound mice, we performed comprehensive immunohistochemical analyses to examine a series of prostatic cellular markers on these high-grade PIN lesions (Fig 5). Atypical cells of PIN lesions failed to immunoreact with Lzts2 (Fig 5B1 and 5B3). Most atypical prostatic cells in the sample of *Lzts2*^*LoxP/LoxP*^ *-Pten*^*loxP/Wt*^:*PB-Cre4* mice showed typical nuclear immunoreactivity with Ar (Fig 5C3), which is similar to the *Pten*^*loxP/Wt*^:*PB-Cre4* mice (Fig 5C2). In samples isolated from both *Pten*^*loxP/Wt*^:*PB-Cre4* and *Lzts2*^*LoxP/LoxP*^ *-Pten*^*loxP/Wt*^:*PB-Cre4* mice, atypical cells showed positive immunoreactivity for E-cadherin and CK8, secretory epithelial markers (Fig 5D2, 5D3, 5E2 and 5E3), but showed no immunoreactivity for the neuroendocrine cell marker, synaptophysin (Fig 5H1–5H3). Immunoreactivity for CK5 and p63, the cellular markers for prostatic basal epithelial cells, appeared mainly in the basal compartment of normal prostatic glands, but rarely in atypical cells in the above mice (Fig 5F2, 5F3, 5G2 and 5G3). Taken together, these data demonstrate that prostatic atypical cells in *Lzts2*^*LoxP/LoxP*^ *-Pten*^*loxP/Wt*^:*PB-Cre4* mainly contain luminal cellular markers.

### Conditional deletion of Lzts2 enhances prostatic cell proliferation and results in alteration of ß-catenin subcellular localization

**Fig 6:**
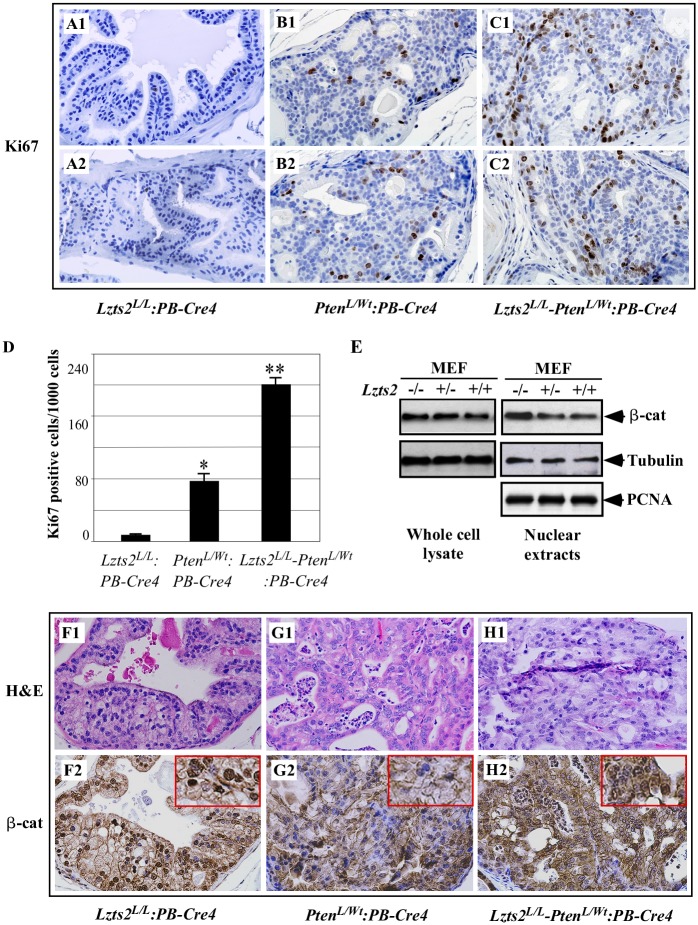
*Lzts2* deletion increases cellular proliferation and nuclear ß-catenin in the mouse prostate. (A-C). Cellular proliferation was examined by immunostaining for Ki-67. Prostate sections isolated from *Lzts2*^*LoxP/LoxP*^:*PB-Cre4*, *Pten*^*loxP/Wt*^:*PB-Cre4*, and *Lzts2*^*LoxP/LoxP*^ *-Pten*^*loxP/Wt*^:*PB-Cre4* mice were stained for Ki-67. (D) A total of 1000 epithelial cells in each lesion from three different lesions from three mice of each genotype were evaluated for Ki-67 immunoreactivity. (E) Mouse embryonic fibroblasts (MEFs) were prepared from different genotype embryos at E10.5. Either whole cell lysates or nuclear extracts were isolated from different genotype MEFs and analyzed by Western-blotting assays for either ß-catenin (ß-cat), tubulin, or PCNA. (F-H) Representative H&E and ß-catenin staining of Prostate tissues from the three different genotype mice is shown. Boxes highlight strong nuclear ß-catenin staining observed with conditional LZTS2 deletion (F2, H2). “*” or “**” means P<0.05 or <0.01, respectively.

It has been shown that deletion of *Pten* enhances proliferation of prostatic epithelial cells in mice [19,27,28]. In this study, we assessed whether deletion of *Lzts2* enhances cell proliferation in the prostate of mice using Ki67 immunohistochemistry. We carefully quantified Ki67 immunostaining in mouse prostate tissues by counting a total of 1000 epithelial cells from five high-power fields in samples isolated from *Lzts2*^*LoxP/LoxP*^:*PB-Cre4*, *Pten*^*loxP/Wt*^:*PB-Cre4*, *and Lzts2*^*LoxP/LoxP*^ *-Pten*^*loxP/Wt*^:*PB-Cre4* mice in different age groups. Experiments were repeated at least three times with three different slides prepared independently in each genotype. As shown in Fig 6A–6D, we presented data prepared from 6–8 month old mice with different genotypes mice. Heterozygous deletion of Pten appears to increases cell proliferation in prostatic epithelial cells in comparison with samples isolated from *Lzts2*^*LoxP/LoxP*^:*PB-Cre4* mice (Fig 6B1 and 6B2 versus Fig 6A1 and 6A2). Intriguingly, a significant increase was observed in Ki67 immunostaining in both mPIN and prostatic adenocarcinoma lesions in *Lzts2*^*LoxP/LoxP*^ *-Pten*^*loxP/Wt*^:*PB-Cre4* compound mice when compared to those in *Pten*^*loxP/Wt*^:*PB-Cre4* mice (Fig 6C1 and 6C2 versus Fig 6B1 and 6B2). The epithelial proliferative index increased from 80 to 240 in HGPIN lesions (P<0.01, Fig 6D). These results demonstrate that *Lzts2* deletion can augment the proliferation of prostatic epithelial cells mediated by *Pten* deletion in the compound mice.

Previously, we have demonstrated that LZTS2 regulates the cellular level and localization of ß-catenin [4]. In this study, we also confirmed the effect of *Lzts2* on cellular ß-catenin in mouse embryonic fibroblasts (MEFs). As shown in Fig 6E, both whole cell lysates and nuclear extracts prepared from different genotypes of MEFs were analyzed for levels of ß-catenin. A notable increase of nuclear ß-catenin was observed in the nuclear extract of *Lzts2* null MEFs despite similar levels of total ß-catenin in whole cell lysates isolated from the same cells. We then performed immunohistochemistry to assess ß-catenin expression in prostate tissues isolated from the three genotypes of mice. We observed typical cell membrane staining of ß-catenin in prostatic luminal cells in the samples isolated from all of three different genotype mice (Fig 6F2, 6G2, and 6H2)., Slightly increased cytoplasmic ß-catenin staining was observed in some of the prostatic epithelial cells of *Pten*^*loxP/Wt*^:*PB-Cre4* mice (Fig 6G2). Intriguingly, a clear nuclear staining of ß-catenin appears in prostatic epithelial cells of samples from *Lzts2*^*LoxP/LoxP*^:*PB-Cre4* and *Lzts2*^*LoxP/LoxP*^ *-Pten*^*loxP/Wt*^:*PB-Cre4* mice (Fig 6F2 and 6H2 boxed). These data further implicate the role of LZTS2 in promoting the nuclear export of ß-catenin in prostatic epithelial cells in mice.

## Discussion

Human *PTEN* and *LZTS2* are localized on the region of 10q23-24, within approximately 15Mb of each other [1]. Loss of heterozygosity (LOH) and homozygous deletions at human chromosomal region 10q23-24 are frequently found in prostate adenocarcinomas, as well as other malignancies, suggesting that multiple tumor suppressors may be present in the region [13]. Most intriguingly, approximately 10% of prostate tumor samples have been shown to possess both *LZTS2* and *PTEN* deletion [29]. In this study, we generated a new mouse model in which both PTEN and LZTS2 were deleted simultaneously in prostatic epithelium to directly assess the biological significance and clinical relevance of PTEN and LZTS2 inactivation in prostate tumorigenesis. As we reported here, we observed accelerated oncogenic transformation and aggressive tumor phenotypes in the prostates of *Lzts2*^*LoxP/LoxP*^ *-Pten*^*loxP/Wt*^:*PB-Cre4* mice with the deletion of both *Pten* and *Lzts2* genes in comparison to *Pten*^*loxP/Wt*^:*PB-Cre4* mice with *Pten* deletion only. Our data demonstrate the biological role of *LZTS2* in tumorigenesis, and implicates the loss of both LZTS2 and PTEN as important biological and relevant events that can directly contribute to prostate cancer development and progression.

Interestingly, similar to humans, both murine *Pten* and *Lzts2* are localized on Chromosome 19, only 11Mb apart from each other [14]. Homozygous deletion of *Pten* in the mouse embryo is lethal and characterized by developmental defects in the mesoderm, endoderm and ectoderm [30]. Heterozygous *Pten* mice develop multiple neoplasia in a wide spectrum of tissues including prostate, thyroid, colon, lymphatic system, mammary gland, and endometrium [30–32]. Conditional inactivation of *Pten* in the murine prostate results in PIN and invasive prostate cancer [19], suggesting a critical role between PTEN inactivation and prostate tumorigenesis. LZTS2 is expressed in testis, prostate, and ovary tissues [4], and reduced expression of LZTS2 transcripts and proteins has been observed in prostate cancer samples [3]. An increase in spontaneous tumor development has been observed in both aged *Lzts2* heterozygous and homozygous knockout mice in comparison to wild type littermates [3]. These heterozygous or homozygous mice also showed an increase of BBN, a carcinogen, induced urinary bladder carcinoma development [3]. These lines of evidence suggest that both PTEN and LZTS2 play critical roles in tumorigenesis, and inactivation of both proteins may have a collaborative effect in oncogenic transformation. Our data presented in this report provide a line of evidence demonstrating combined loss of LZTS2 and PTEN as an important biological event in prostate cancer development and progression.

Multiple lines of evidence suggest that the *Lzts2* gene is a tumor susceptibility gene [3]. Our previous data also showed a potential role of Lzts2 in prostate tumorigenesis. In this study, we also generated mice with conditional inactivation of Lzts2 in prostatic luminal epithelial cells using PB-Cre transgenic mice to directly examine Lzts2 in prostate tumorigenesis, [15]. We did not observe significant pathological changes in the prostate of both *Lzts2*^*LoxP/wt*^:*PB-Cre4* and *Lzts2*^*LoxP/LoxP*^:*PB-Cre4* mice up to 20-months of age (data not shown). These results imply that selective inactivation of Lzts2 in prostatic luminal epithelial cells by the ARR2PB promoter is insufficient to induce oncogenic transformation in prostatic luminal epithelial cells [15]. Homozygous deletion of *Pten* in the murine prostate results in invasive prostate cancer and metastatic prostate cancer of the lymph nodes and lung as early as ages of 2-months [19]. However, conditional heterozygous inactivation of *Pten* in the mouse prostate showed slow and moderate PIN and prostatic adenocarcinomas development [19]. Therefore, we used *Lzts2*^*LoxP/LoxP*^ *-Pten*^*loxP/Wt*^:*PB-Cre4* compound mice to further evaluate the combined effect of Lzts2 and Pten inactivation in the prostate of mice. As detailed in this study, homozygous inactivation of Lzts2 in the mouse prostate accelerates the oncogenic transformation mediated by heterozygous loss of *Pten* in prostatic luminal epithelial cells. Given that *PTEN* loss of heterozygosity has been frequently observed in human tumors, *Lzts2*^*LoxP/LoxP*^ *-Pten*^*loxP/Wt*^:*PB-Cre4* mice may mimic what occurs during the course of human prostate cancer development, and can be used to characterize this mechanism of prostate cancer initiation and progression. Specifically, identification of possible pathways and molecules that are involved in Lzts2 and Pten mediated tumorigenesis using the above mouse models would be biologically significant and clinical relevant.

Dysregulation of Wnt and ß-catenin mediated signaling pathways events in the pathogenesis of variety of human malignancies, including prostate cancer [33,34]. It has been shown that tumor cells contain high levels of nuclear ß-catenin through different regulatory mechanisms [35]. LZTS2 has been demonstrated to regulate ß-catenin nuclear export and modulate its cellular distribution and activity [4]. In this study, using *Lzts2*-deleted MEFs, we also assessed the effect of Lzts2 on the cellular localization of ß-catenin. Although we observed almost equal levels of ß-catenin in whole cell lysates prepared from either wild type or heterozygous and homozygous *Lzts2* deletion MEFs, a significant increase of nuclear ß-catenin appears in *Lzts2* null MEFs. This observation is consistent with previous data and demonstrates an important role of Lzts2 in regulating ß-catenin nuclear export [4]. PTEN exerts its function as a tumor suppressor through negative regulation of PI3K/AKT signaling pathways [5]. PI3K/Akt increases the stability of nuclear ß-catenin by phosphorylation and inactivation of the downstream substrate, GSK3ß, in prostate cancer cells, and PTEN deletion can augment PI3K/AKT action and increase cellular ß-catenin [10]. As shown in this study, prostate cancer cells co-transfected with both wild type PTEN and LZTS2 expression vectors showed less transcriptional activity of Tcf/ß-catenin than those transfected with either PTEN or LZTS2 alone. Interestingly, PTEN expression showed a much stronger inhibitory effect on wild type of ß-catenin than mutated ß-catenin. In contrast, LZTS2 expression inhibits both wild type and mutated ß-catenin activity. Through these distinct mechanisms, PTEN and LZTS2 collaboratively regulate cellular levels of ß-catenin and act as tumor suppressors to inhibit Wnt/ß-catenin-mediated oncogenic transformation in cells. In addition, we observed an increase in PIN and prostatic tumor development in *Lzts2*^*LoxP/LoxP*^ *-Pten*^*loxP/Wt*^:*PB-Cre4* compound mice in comparison to *Pten*^*loxP/Wt*^:*PB-Cre4* mice. Most atypical and tumor cells in *Lzts2*^*LoxP/LoxP*^*-Pten*^*loxP/Wt*^:*PB-Cre4* mice appear to be E-cadherin and CK8 positive, suggesting that they are of luminal epithelial cellular origin. In this study, we also measured cell proliferation in samples isolated from different mice. Prostatic luminal cells isolated from *Lzts2*^*LoxP/LoxP*^*-Pten*^*loxP/Wt*^:*PB-Cre4* compound mice appear more proliferative than those from other genotypes of mice. We also observed more cellular ß-catenin expression in atypical and tumor cells in the prostate of *Pten*^*loxP/Wt*^:*PB-Cre4*, and *Lzts2*^*LoxP/LoxP*^*-Pten*^*loxP/Wt*^:*PB-Cre4* mice. Interestingly, deletion of *Lzts2* alone showed more nuclear ß-catenin expression than the other genotypes in the above samples. These data provide a link between increased cellular ß-catenin and oncogenic transformation in prostatic luminal epithelial cells. Validation of PTEN and LZTS2 loss, as well as cellular ß-catenin expression and localization within human tumor samples will provide useful information about the roles of PTEN and LZTS2 in human tumorigenesis; this knowledge may lead to the development of new therapeutic strategies for prostate cancer and other human malignancies.
